# Characterization of a Novel Acyl-ACP Δ^9^ Desaturase Gene Responsible for Palmitoleic Acid Accumulation in a Diatom *Phaeodactylum tricornutum*

**DOI:** 10.3389/fmicb.2020.584589

**Published:** 2020-12-16

**Authors:** Baoling Liu, Yan Sun, Wei Hang, Xiaodan Wang, Jinai Xue, Ruiyan Ma, Xiaoyun Jia, Runzhi Li

**Affiliations:** ^1^College of Agriculture, Shanxi Agricultural University, Jinzhong, China; ^2^College of Plant Protection, Shanxi Agricultural University, Jinzhong, China

**Keywords:** *Phaeodactylum tricornutum*, palmitoleic acid, acyl-ACP Δ^9^ desaturase, substrate selectivity, oil biosynthesis and regulation

## Abstract

Palmitoleic acid (16:1Δ^9^) possesses a double bond at the seventh carbon atom from methyl end of the acyl chain and belongs to unusual ω-7 monounsaturated fatty acids with broad applications in food, pharmaceuticals, cosmetics, biofuel, and other industries. This high-value fatty acid accumulates up to >40% of total lipid in the marine diatom *Phaeodactylum tricornutum.* The present study was conducted to determine the key gene responsible for 16:1Δ^9^ biosynthesis in this unicellular alga. A new full-length cDNA and genomic DNA encoding acyl-ACP Δ^9^ desaturase (PtAAD) were isolated from *P. tricornutum* cells. Expression levels of *PtAAD* gene under normal and stress culture conditions were both positively correlated with 16:1Δ^9^ accumulation, implying its potential role for fatty acid determination. Functional complementation assay of a yeast mutant strain *BY4839* evidenced that *PtAAD* could restore the synthesis of unsaturated fatty acid, especially generating high levels of 16:1Δ^9^. Further transient expression of *PtAAD* gene in *Nicotiana benthamiana* leaves was accompanied by the accumulation of 16:1Δ^9^, which was absent from control groups. Three-dimensional structure modeling studies showed that functional domain of PtAAD contained three variant amino acids (F160, A223, and L156), which may narrow the space shape of substrate-binding cavity to ensure the entry of 16:0-ACP. Consistent with this prediction, the mutated version of *PtAAD* gene (F160L, A223T, and L156M) in *N. benthamiana* systems failed to accumulate 16:1Δ^9^, but increased levels of 18:1Δ^9^. Taken together, PtAAD exhibits a strong enzymatic activity and substrate preference for 16:0-ACP, acting as the key player for high biosynthesis and accumulation of 16:1Δ^9^ in this alga. These findings provide new insights for better understanding the palmitoleic acid and oil biosynthetic mechanism in *P. tricornutum*, indicating that *PtAAD* gene may have practical applications for enriching palmitoleic acid and oil yield in other commercial oleaginous algae and crops.

## Introduction

Palmitoleic acid (16:1Δ^9^), a kind of monounsaturated ω-7 fatty acid, possesses a double bond at the seventh carbon atom starting from the methyl end of the acyl chain (Bryant et al., 2016). It is initially biosynthesized by a variant acyl-ACP desaturase known as Δ^9^-16:0-ACP desaturase, using saturated palmitic acid (16:0) as a substrate (Wu et al., 2011; Nguyen et al., 2015). Palmitoleic acid has important values for human health and industrial applications. It is widely used as a health-benefit food, promoting cardiovascular fitness, improving human immunity, and inhibiting tumorigenesis (Akazawa et al., 2010; Wu et al., 2012; Nunes and Rafacho, 2017; De Souza et al., 2018). It is also an ideal material to produce polyethylene and excellent-quality biofuels in industry (Michael et al., 2010; Nguyen et al., 2015; Wang et al., 2018a).

Unfortunately, this valuable fatty acid is not typically abundant in common oilseed crops, such as soybean, canola, cotton, and peanut. This fatty acid is only accumulated largely in several wild plants including cat’s claw (*Doxantha unguis-cati* L.), macadamia (*Macadamia* sp.), and sea buckthorn (*Hippophae rhamnoides*) with content of >64%, ∼30%, and ∼32%, respectively (Wu et al., 2012; Ding et al., 2019). However, the poor agronomic traits (small seeds, low yield, and narrow distribution) greatly limit the commercial production of palmitoleic acid derived from those plants (Nguyen et al., 2015; Ding et al., 2019). Recently, rising cost of palmitoleic acid together with increased market need have led to an unprecedented contradiction between supply and demand (Nguyen et al., 2015). Therefore, it is urgent to develop a new way to increase production scale for commercial utilization of palmitoleic acid. *Phaeodactylum tricornutum*, a type of unicellular eukaryotic diatom, could offer a suitable platform to commercially produce palmitoleic acid because this alga is able to accumulate as high as 46% palmitoleic acid among total lipids (Gruber et al., 2015; Yang et al., 2017; Zulu et al., 2018). Most importantly, this alga shows rapid growth, strong environmental adaptability as well as huge biomass under liquid culture condition, which make it possible to cultivate this alga in a large scale for commercial production of palmitoleic acid without occupying more farmland (Dolch and Maréchal, 2015; Lina-Juana and Eric, 2015; Li and Ismar, 2018; Zulu et al., 2018). Undoubtedly, *P. tricornutum* is an ideal resource to develop 16:1Δ^9^-based nutraceuticals and excellent industrial products, rather than traditional applications for EPA, DHA, and fucoxanthin (Domergue et al., 2003; Hamilton et al., 2014; Patel et al., 2019). So far, it is still unclear how palmitoleic acid is highly biosynthesized and accumulated in this alga.

In plants, *de novo* synthesis of 16C or 18C saturated fatty acids occurs in plastids by a series of fatty acid synthases (Harwood, 1996). Stromal acyl-ACP Δ^9^ desaturases (AADs, EC: 1.14.99.6) play crucial roles in the first desaturation of fatty acid, introducing a double bond into saturated acyl chains to form the first monoenes (Lindqvist et al., 1996). Some AAD isoforms can select and catalyze their specific substrates to generate distinct monoenes with different unsaturation position within aliphatic chains (Troncoso-Ponce et al., 2016). For example, stearoyl-ACP Δ^9^ desaturases (SADs) desaturate 18:0-ACP to form 18:1Δ^9^-ACP (oleic acid) at high efficiency. SADs are the predominant AAD isoforms in most of the oil plants. Correspondingly, the majority of the FAs in seed oil comprise oleic acid as well as its derivatives (linoleic acid, linolenic acid, and so on). However, a few AAD isoforms prefer to choose 16:0-ACP as substrate, instead of 18:0-ACP. Such AAD isoforms were denoted as palmitoyl-ACP Δ^9^ desaturases (PADs). PADs specifically catalyze biosynthesis of ω-7 palmitoleic acid (16:1Δ^9^), which can be further elongated to form other ω-7 fatty acids, 18:1Δ^11^ and 20:1Δ^13^ (Lindqvist et al., 1996; Behrouzian et al., 2002; Guy et al., 2011). Although some genes encoding PADs were identified from a few higher plant species rich in ω-7 fatty acids (Cahoon et al., 1998; Bryant et al., 2016; Liu et al., 2019), little knowledge is available for this type of gene and its functions as well as the biosynthesis and regulation mechanism in *P. tricornutum*, a high accumulator of palmitoleic acid.

Therefore, the main objective of the present study was to identify *P. tricornutum* gene encoding acyl-ACP Δ^9^ desaturase (*PtAAD*) using genome approach. Furthermore, functions of PtAAD, particularly enzymatic activity and substrate specificity, were characterized by yeast function complementary assay and transient expression in *Nicotiana benthamiana* leaves. The existence of three variant amino acid residues might determine the specific catalytic property of the PtAAD reaction. Dynamic of palmitoleic acid accumulation was also examined in algal cells cultivated under normal and stress conditions. Such investigations could generate new knowledge for understanding palmitoleic acid biosynthesis and regulation mechanism in algae, providing scientific references to optimize culture conditions and genetic engineering of this alga for increasing palmitoleic acid yield and its market utilization.

## Materials and Methods

### Algal Strain and Culture Conditions

*Phaeodactylum tricornutum* was purchased from Chinese Freshwater Algae Resource Center, Institute of Hydrobiology, Chinese Academy of Sciences. The algal strain at log phase was inoculated into sterilized 250 ml f/2 liquid medium (75 g/L NaNO_3_) at a ratio of 1:100. The cultivation conditions were set as 200 μmol photons m^–2^ s^–1^ for light intensity, 12 h light/12 h dark for photoperiod, and 25°C for temperature in illuminating incubator (Li et al., 2018; Wang et al., 2018b; Cui et al., 2019a).

When the initial algae concentration was controlled at OD_680_ ≈ 80 n, the nitrogen stress of 37.5 g/L NaNO_3_ replaced the previous amount of 75 g/L as the treated group, namely 1/2N. The algae cultured with 75 g/L NaNO_3_ was regarded as the control group (N^+^). In the process of cultivation, flasks were shaken several times in the morning and evening, and OD_680_ was detected on the fixed time point every day with three repetitions until the 10th day. In this study, all the algal cells (N^+^ and 1/2N) cultured for 0, 5, and 10 days were collected by centrifugation at 5,000 rpm for 10 min (Guihéneuf et al., 2011). Then, the algal cells washed with deionized water were immediately frozen in liquid nitrogen and stored in -80°C for subsequent experiments.

### Gene Identification, Functional Domain, and Three-Dimensional Modeling Analysis

Since the complete genome sequence of *P. tricornutum* has been released (Bowler et al., 2008), an acyl-ACP Δ^9^ desaturase gene sequence of *CrSAD* (EDP04705.1) from *Chlamydomonas reinhardtii* was used as query to blast by setting the *E*-value of 1e^–10^ against *P. tricornutum* genome database^1^ (Bowler et al., 2008). The conserved domain of acyl-ACP Δ^9^ desaturase was verified and the protein was then identified as the PtAAD by analysis using HMMER version 3.0, SMART website^2^ and CDD website^3^. All parameters were default for these online examinations.

The coding region of *PtAAD* gene was predicted by ORF Finder^4^. Gene Structure Display Server 2.0 (GSDS)^5^ was used to analyze the gene structure. ExPASy-ProtParam^6^ was taken to calculate the molecular weight and isoelectric point. TargetP 1.1 Server^7^ and ChloroP^8^ were employed to predict the subcellular localization of this enzyme protein.

The protein sequences of AtFAB2 (AT2G43710) and AtAAD3 (AT5G16230) from *Arabidopsis thaliana* were obtained from TAIR database^9^ (Lightner et al., 1994; Troncoso-Ponce et al., 2016). Peptide sequences from castor (RcSAD1, NP_001310659.1) (Lindqvist et al., 1996) and cat’s claw (Muc-PAD, AAC05293) were downloaded from NCBI^10^ (Cahoon et al., 1998). All amino acid sequences including PtAAD and reference templates (RcSAD1, AtFAB2, AtAAD3, and Muc-PAD) were aligned by GenDoc software to identify the key amino acid residues which influence the catalytic properties in functional domain. Three-dimensional structure of PtAAD was modeled by Swiss-model^11^ where the crystal structure of RcSAD1 was used as the template. In addition, the key amino acids and ligands in catalytic activity center were marked by Discovery Studio 4.1 software with default parameters.

### RNA Extraction and qRT-PCR Assays

The frozen *P. tricornutum* cell samples were grinded in liquid nitrogen to extract total RNA and then reversely transcripted into the first strand of cDNA according to Aidlab manufacturer’s instructions (Aidlab, Beijing, China). Specific primers for real-time PCR were designed to detect mRNA levels of *PtAAD* gene from different samples at different nitrogen stress days. Then, qRT-PCR was conducted by Bio-Rad system (CFX96). The 20-μl reaction system (ABM Biotech, Zhenjiang, China) contained 10 μl EvaGreen Mix (2×), 1 μl cDNA (50 ng), 7.8 μl nuclease-free H_2_O, and F/R primers of 10 μmol/L with 0.6 μl, respectively. The PCR program was at 95°C of pre-denaturation for 30 s, followed by 40 cycles which comprised 95°C for 15 s in denaturation process, and 58 and 72°C for 30 s at annealing and extending stages, respectively. Each reaction and treatment was set in three replicates. Relative expression values were tallied and calculated with the method of 2^–ΔΔCt^ by SPSS 17.0 software (Livak and Schmittgen, 2001). The corresponding primers of *PtAAD* and internal reference gene (*Ptactin*) are listed in Supplementary Table 1.

### Cloning of *PtAAD* Gene and Recombinant Vector Construction

The cDNAs of algal cells cultured for 10 days in 1/2 nitrogen stress were used as the template for PCR cloning. The complete open reading frame (ORF) of putative *PtAAD* gene was cloned by PtAAD-F and PtAAD-R with restriction site of *Xba*I/*Kpn*I (Supplementary Table 1). The 20-μl amplification system consisted of 1.0 μl cDNA, 10 μl 2 × Taq PCR Master Mix, 2.0 μl of forward and reverse primers, and 7 μl nuclease-free ddH_2_O (ABM Biotech, Jiangsu, China). The reaction program was set according to the following procedure: pre-degeneration at 95°C for 5 min, then followed by 35 cycles containing denaturation at 94°C for 1 min, annealing at 58°C for 1 min, and extension at 72°C for 90 s and final 10-min extension at 72°C. The PCR product was ligated into a pEASY-blunt Zero cloning vector (TransGen Biotech, Beijing, China) and then sequenced after a series of procedures including gel extraction, purification, and transformation into *Escherichia coli* strain (DH5α). Finally, the amplified fragment was digested with *Xba*I and *Kpn*I. The digested fragment was constructed into the corresponding sites of plant expression vector of pCAMBIA1303 containing CaMV 35S promoter (Invitrogen) by T_4_ DNA ligase to form the recombinant plasmid of Pro35S:PtAAD. The positive Pro35S:PtAAD plasmid was transformed into *Agrobacterium tumefaciens* GV3101 to perform follow-up infestation experiments. The mutated version of *PtAAD* gene (*PtAAD-M*) was created by direct gene synthesis (synthesized by Sangon, China).

For yeast expression study, pYES2 vector was used for the construction of the target gene expression vector, which comprised promoter *GAL1* allowing target gene expression induced by galactose and selective marker *URA3* for positive clone growth on lack uracil medium. The ORF sequence of *PtAAD* and gene was amplified from clone vector and then inserted into pYES2 vector to form the recombinant pYES2-PtAAD by *Hin*dIII/*Xba*I after codons were optimized for yeast preference. The subsequent transformation in *E. coli* and verification procedures were similar to the aforementioned operations.

### Transient Expression of *PtAAD* and *PtAAD-M* Gene in *N. benthamiana* Leaves

Wild-type *N. benthamiana* plants were grown at about 26°C under greenhouse conditions with 16 h/8 h natural photophase and 60% relative humidity (Zhang et al., 2014). The healthy seedlings were selected for infection when cultured for 6 weeks.

*A. tumefaciens* GV3101 containing p1303-PtAAD and p1303-PtAAD-M was cultured at 28°C overnight. When the concentration was up to OD_600_ ≈ 00 c, the bacteria were centrifuged at 12,000 rpm to collect cell pellets. Before infiltration, the bacteria were suspended with sterilized ddH_2_O containing 200 μmol/L acetosyringone, 10 mM/L MgCl_2_, and 10 mM/L MES, keeping the final concentration at OD_600_ ≈ 0.2.

Then, *Agrobacterium* infiltration was operated with the method as described Liu et al. (2019). The developing leaves of *N. benthamiana* were selected for infiltration with well-suspended *Agrobacterium*. A needle was used to prick a wound on the back of the leaf along the longitudinal axis, and then the *Agrobacterium* infiltrated an area of about 1 cm diameter. Half of the leaf was injected with the empty vector, and the other half was infected by the recombinant plasmid. Finally, the treated seedlings were normally cultured for 5 days and then the infected parts of the leaves were freeze-dried for preparation of fatty acid methyl esters.

### Yeast Culture and Heterologous Expression of *PtAAD* in the Unsaturated Fatty Acid-Defective *S. cerevisiae* Mutant *BY4389*

The *Saccharomyces cerevisiae* mutant strain *BY4389* (His^–^, Leu^–^, and Ura^–^), unable to synthesize unsaturated fatty acids because of *OLE1* mutation, was initially purchased from Osaka University, Osaka, Japan (Xue et al., 2016). The strain was initially cultured at 30°C in YPD medium consisting of 2% glucose, 2% peptone, 1% yeast extract, and extra 0.01% linoleic acid at a speed of 250 rpm. Then the cells were cooled and collected by centrifugation at 8,000 rpm for 10 min when the content was up to OD_600_ ≈ 0.8 (Liu et al., 2019). The yeast cells were suspended in ice-cold 100 mM LiAc to final concentration of 2 × 10^9^ cells ml^–1^ and dispensed into 50 μl per 1.5-ml tube as receptor cells for transformation after being washed three times with precooling sterilized ddH_2_O.

For transformation, the receptor cells were added into 360 μl mixed solution containing 1 μg pYES2-PtAAD recombinant plasmid, 240 μl 50% PEG, 36 μl 1 M LiAc, 5 μl 10 mg/ml carrier DNA, and sterile ddH_2_O according to the protocol (Coolaber, Beijing, China). The mixed solution was successively kept at 30 and 42°C for 30 min, respectively. In addition, the empty vector pYES2 was used as blank control. The yeast transformants were subsequently grown on sc-ura medium (lack uracil, 0.01% linoleic acid included) in which only positive yeasts could grow.

The positive transgenic yeasts were cultured in sc-ura medium containing 0.01% linoleic acid, keeping fast growth for more cells. After that, glucose (2%, *w*/*v*) was replaced by galactose (2%, *w*/*v*) and linoleic acid was removed to induce yeast cells for another 72 h at 26°C and 150 rpm on a shaker. Finally, the induced cells were collected by centrifugation and freeze dried for subsequent fatty acid extraction. In addition, the transgenic cells were inoculated in the induced medium containing 2% galactose (without uracil and any unsaturated fatty acids) to detect whether the transgenic cells can survive and produce unsaturated fatty acids.

### Lipids Preparation and Gas Chromatographic (GC) Analysis

Total lipids of algal cells were extracted and tested based on cell dry weight as previously described (Radakovits et al., 2011). Samples from *P. tricornutum* cells, *N. benthamiana* leaves, and transgenic yeast were grinded into powder and dissolved with chloroform/methanol (2:1, *v*/*v*) to extract total lipids as previously described (Bhattacharya et al., 2015). Total lipid proportion (L,% *w*/*w*) was calculated by equation L = W1/W2 where W1 and W2 represent total lipid content (mg/ml) and cell dry weight concentration (mg/ml), respectively.

Then, fatty acid methyl esters (FAMEs) were further extracted by esterification process with sulfuric acid/methanol solution (2.5%, *v*/*v*) as previously described (Liu et al., 2019). C17:0 was used as internal standard. Finally, FAMEs were analyzed by Agilent 7890B gas chromatograph of which parameters and detailed running procedures were previously described (Liu et al., 2019).

## Results

### Nitrogen Stress Significantly Promoted Palmitoleic Acid Biosynthesis in *P. tricornutum*

**FIGURE 1:**
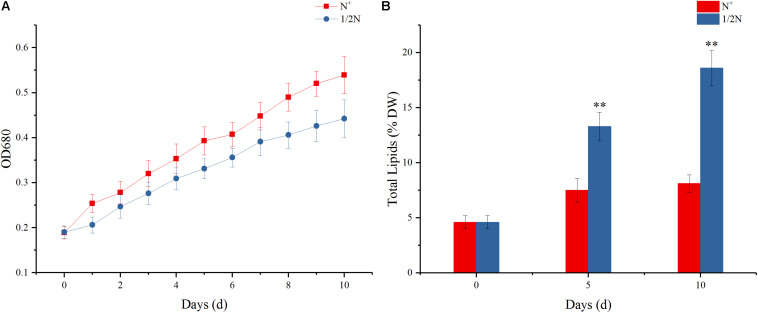
**(A)** Growth curve from 0 to 10 days of *Phaeodactylum tricornutum*. OD_680_ showed the cell density indicating the growth rate. **(B)** Total lipid content at 0, 5, and 10 days of *P. tricornutum* cultured in nitrogen-deficient and -sufficient medium. Values are the means ± SE of six biological duplicates. **p < 0.01.

Previous reports showed that nitrogen stress can limit the growth of algal cells and trigger TAG accumulation (Abida et al., 2015). To explore the accumulation pattern of palmitoleic acid in response to stress, the growth parameter and total fatty acid contents in algal cells were examined under nitrogen-normal (N^+^) and nitrogen-stressed (1/2N) conditions (Figure 1 and Supplementary Table 2). Compared with the control group (N^+^), algal cell density in the treated group (1/2N) obviously decreased, whereas total lipid contents were increased to 13.3 and 18.6% of dry weight at the 5th and 10th days, respectively (Figures 1A,B). This means that nitrogen seriously affects algal growth rate and lipid accumulation, which is consistent with previous studies (Abida et al., 2015; Cui et al., 2019a). Yang et al. (2014a, b) also indicated that more energy and carbon flux used in photosynthesis and biosynthesis of amino acids were redirected to accumulate lipids under nitrogen stress.

In addition, fatty acid profiling of all algal cells cultured for 5 and 10 days (Supplementary Table 2) showed that compared with N^+^ group, contents of total saturated and monounsaturated fatty acids in 1/2N group both significantly enhanced with the increase of cultivation days, whereas the accumulation of polyunsaturated fatty acids was decreased. It is noteworthy that the 16:1Δ^9^ levels in algal cells cultured in 1/2N medium for 5 and 10 days were 40.25 and 44.61%, far higher than 32.74 and 33.56% in N^+^ medium, respectively. At the same time, content of 18:1Δ^11^, the extension product of 16:1Δ^9^, also greatly increased during the whole culture process. Overall, the fatty acid profiles indicated that nitrogen stress may induce the transcriptional expression and regulation of the related gene, which further increase the pathway of catalyzing palmitic acid (16:0) to form palmitoleic acid (16:1Δ^9^) and simultaneously affect the accumulation of other unsaturated fatty acids (e.g., EPA).

Moreover, the protein 3D structures were predicted by Swiss-model to display the relative positions between the key amino acids and diiron catalytic centers of PtAAD and the temples of AtFAB2 and AtAAD3 with the well-characterized space structure (Figure 5). The three-dimensional configuration showed that the eight key amino acids were located near the side and bottom of the substrate-binding cavity and close to the diiron center of the enzymes examined (Figures 5I,II). The divergent AAs in the function domain of PtAAD were similar to that of AtAAD3. Also, these variant AAs were exactly located in the bottom of the substrate binding cavity, regardless of their types and numbers, suggesting that they significantly make the substrate-binding channel of PtAAD short to prefer 16:0-ACP but not 18:0-ACP (Figures 5II–D).

### Cloning and Expression of *PtAAD* Gene Under Nitrogen Stress Condition

**FIGURE 2:**
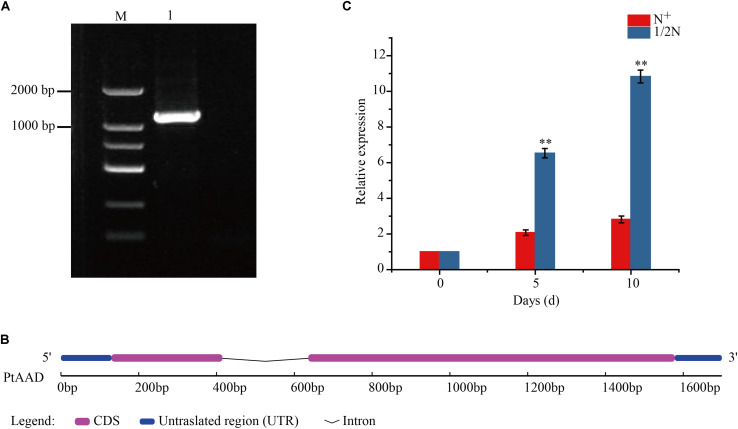
**(A)** The amplified fragment of *PtAAD* gene. M: D2000 marker; lane 1: *PtAAD* gene. **(B)** Schematic illustration of *PtAAD* gene structure. Pink boxes: extrons. Gray line: intron. Blue boxes: untranslated region (UTR). **(C)** Expression patterns of *PtAAD* gene at 0, 5, and 10 days on nitrogen-stressed condition. Bar charts show the relative expression levels of *PtAAD* gene normalized to that of *Ptactin* measured by qRT-PCR. The analysis was performed with three biological samples for each tissue. The method of 2^– ΔΔCt^ was used in this analysis. “*” and “**” indicate *p* < 0.05 and *p* < 0.01, respectively.

To explore the key gene responsible for biosynthesis of palmitoleic acid, the CrSAD sequence was used to blast and search in *P. tricornutum* genome database so as to identify homolog genes encoding AAD enzyme, which can catalyze 16:0 to form 16:1Δ^9^. One candidate acyl-ACP Δ^9^ desaturase gene sequence was identified in *P. tricornutum* genome, namely *PtAAD.* The complete ORF in length of 1,227 bp was successfully cloned by PCR using the template cDNA from algal cells cultured under 1/2 N stress condition (Figure 2A and Supplementary Table 3). The *PtAAD* genomic sequence was 1,699 bp long, consisting of one intron, two exons, and 5’ and 3’ untranslated regions (Figure 2B). It was worthy to note that *Phat3_J9316* described by Dolch and Maréchal (2015) showed high sequence homology with *PtAAD* identified here. However, *Phat3_J9316* did not contain a complete ORF. As shown in Supplementary Table 3, the length of *Phat3_J9316* sequence is 300 bp shorter than that of the *PtAAD* sequence.

Subcellular localization predicted that the deduced PtAAD protein (408 amino acids, AAs) had a 39-AA chloroplast transit peptide and was located in chloroplast with scores of 0.7852. The calculated molecular mass and theoretical isoelectric point of PtAAD protein were 46.36 kDa and 5.19, respectively.

**FIGURE 3:**
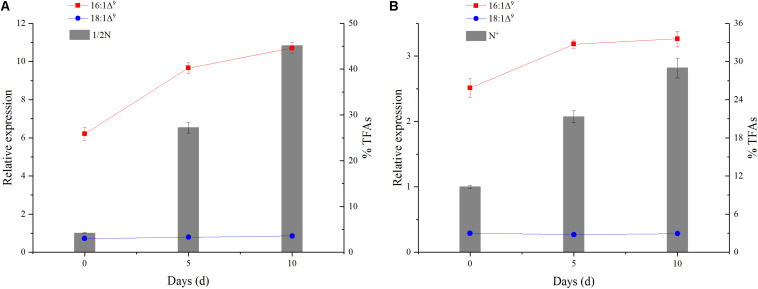
The correlation of the expression of *PtAAD* with molar percentage of C16:1Δ^9^ and C18:1Δ^9^ under nitrogen-depleted condition **(A)** and normal culturation condition **(B)**. Columns show percentage of total fatty acid composition and line for relative expression of *PtAAD*. Three biological replicates were analyzed in each tissue.

Expression analysis revealed that *PtAAD* transcript was increased by 3.2- and 3.9-fold higher in 1/2N group than that in N^+^ group on the 5th and 10th days, respectively (Figure 2C). In addition, the relative expression levels of *PtAAD* gene presented a notably positive correlation with the accumulation of 16:1Δ^9^ with correlation coefficient of *r*^2^ = 0.965 in 1/2N group (Figure 3A) and *r*^2^ = 0.928 in N^+^ group, respectively (Figure 3B). However, the expression pattern of *PtAAD* showed inconsistent relevance with the accumulation of 18:1Δ^9^ with *r*^2^ = 0.702 in 1/2N group (Figure 3A) and *r*^2^ = -0.105 in N^+^ group (Figure 3B). These results indicated that *PtAAD* gene perhaps mainly participated in the biosynthesis of palmitoleic acid rather than oleic acid.

### Analysis of Key Amino Acids Determining Substrate Specificity of PtAAD Protein

**FIGURE 4:**
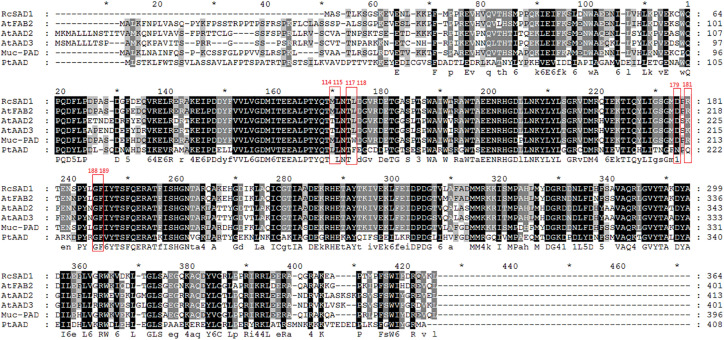
Multiple sequence alignment of PtAAD protein sequence and other temples (RcSAD1, AtFAB2, AtAAD3, and Muc-PAD). The GenBank or TAIR accession numbers of the temple sequences are listed as follows: RcSAD1 (NP_001310659.1) from *Ricinus communis* (Whittle and Shanklin, 2001); AtFAB2 (At2g43710), AtAAD2 (At3g02610), and AtAAD3 (At5g16230) from *A. thaliana* (Troncoso-Ponce et al., 2016); Muc-PAD (AFV61670.1) from *D. unguis-cati* (Cahoon et al., 1998). Red box represents key amino acids. Red numbers represent corresponding position of residues from castor.

To investigate whether PtAAD enzyme has the specific catalytic activity for 16:0-ACP, protein sequence of PtAAD was aligned with the well-characterized AADs from other plants including 18:0-ACP-specific (RcSAD1 and AtFAB2) and 16:0-ACP-specific (AtAAD3, AtAAD2, and Muc-PAD) enzymes to identify the conserved function domain (Figure 4 and Table 1). Remarkably, the eight key amino acids in the conserved function domain are identified for 18:0-ACP-specific RcSAD1 and AtFAB2. However, one or three of the eight key amino acids were variants for 16:0-ACP-specific isoforms AtAAD2/3 and Muc-PAD. Three variant AA residues (F160, A223, and L156) of the eight key amino acids were also present in the domain of PtAAD (Figure 4 and Table 1), suggesting that PtAAD may have the substrate specificity for 16:0-ACP.

**TABLE 1:** Key amino acids in the functional domain of PtAAD protein and other AADs.

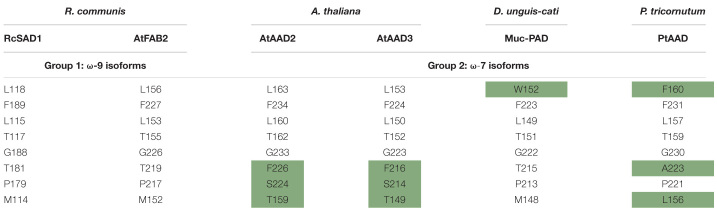	

*Shaded cells: variant amino acids in the domain of 16:0-ACP-specific isoforms and PtAAD, respectively, compared with 18:0-ACP-specific isoforms of RcSAD1 and AtFAB2 in the conserved function domain.*

### Transient Expression of *PtAAD* and *PtAAD-M* Gene in *N. benthamiana* Leaves

**FIGURE 5:**
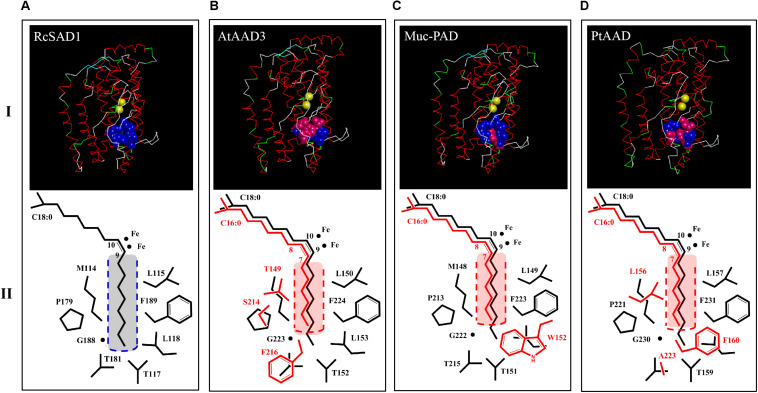
3D structure models of PtAAD protein monomer [**(D)**, upper panel I] and C18:0/C16:0-ACP chains as well as side chains of key amino acids close to catalytic center of diiron ions (II lower panel). All 3D models were predicted on Swiss-model (https://swissmodel.expasy.org/) with RcSAD1 (NP_001310659.1) and AtAAD3 as temple **(A,B)**. RcSAD1 (NP_001310659), AtAAD3 (At5g16230) and Muc-PAD (AFV61670.1) as templates **(A–C)** File of 3D structure of PtSAD is shown in Discovery Studio 4.1 software. Blue bolls/spheres in the upper panel represent common amino acids that were the same as RcSAD1 while pink bolls show the varied amino acids. The fatty-acyl chains and side chains of amino acids were drawn by ChemDraw software (II panel). Black color represents C18:0-ACP and its amino acids. Red indicates C16:0-ACP and varied amino acids and yellow bolls for diiron ions. Black and red dashed boxes stand for substrate binding cavity (or channel) of 18:0-ACP and C16:0-ACP, respectively (II panel).

**FIGURE 6:**
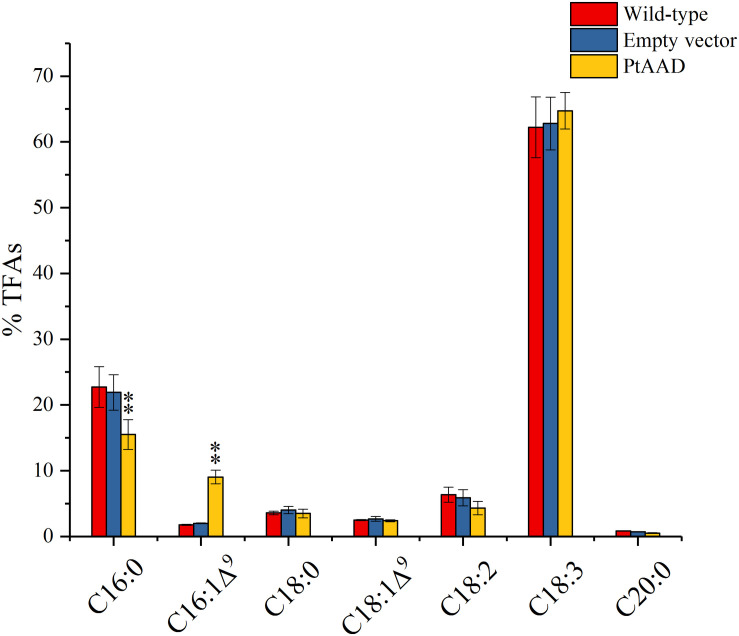
Fatty acid composition in *N. benthamiana* leaves transiently expressing *PtAAD* gene. Fatty acids were analyzed by GC and the data are means of mol% ± SE with three independent replicates. “*” and “**” indicate p < 0.05 and p < 0.01, respectively. Empty vector of pCAMBIA1303 was used as negative control. Data are shown as mean ± SE (*n* = 3).

To further examine the catalytic specificity of PtAAD for 16:0-ACP, the transient overexpression assay of *PtAAD* was performed in *N. benthamiana* leaves mediated by *Agrobacterium* infiltration. The empty vector of pCAMBIA1303 and the uninfected leaves were used as negative and blank controls, respectively. The infected leaves were collected at the sixth day after infiltration and prepared for lipid extraction. FAME analysis by GC showed that the leaves overexpressing *PtAAD* gene produced large amounts of palmitoleic acid (16:1Δ^9^) by 9.04% higher than that in the blank and negative controls (Figure 5), whereas oleic acid (18:1Δ^9^) level was not evidently increased. In contrast, the content of palmitic acid (16:0) significantly decreased by 15.5% compared with the controls (Figure 6). These results evidenced that PtAAD can specifically catalyze 16:0 to form 16:1Δ^9^. In addition, contents of stearic acid (18:0), polyunsaturated linoleic acid (18:2Δ^9,12^), and linolenic acid (18:3Δ^9,12,15^) had slight changes in *PtAAD*-expressed leaves despite the statistical insignificance, suggesting that the activity of the related endogenous enzymes might be little affected by the foreign gene expressed in these tobacco leaves. To test the influence of the aforementioned AA difference on the catalyzed property of PtAAD, we also created a mutated version of *PtAAD* gene (*PtAAD-M*) by direct gene synthesis (F160L, A223T, and L156M). Consistent with the 3D structure prediction, transient overexpression of *PtAAD-M* gene in *N. benthamiana* leaves failed to accumulate 16:1Δ^9^, but increased levels of 18:1Δ^9^ (Supplementary Figure 1).

To verify the prediction that PtAAD is a soluble enzyme protein localized in chloroplasts, we constructed the recombinant protein expression vector of 35S:PtAAD-GFP and then introduced into tobacco leaf tissues by *Agrobacterium-*mediated infiltration. The mesophyll cells were isolated, and protoplasts were observed using a laser scanning confocal microscope. As shown in Supplementary Figure 2, the strong green fluorescence occurred in the chloroplast, showing that PtAAD was localized in the chloroplast when *PtAAD* was heterologously expressed in tobacco leaf tissues. This plastid-localized feature detected for PtAAD further supports that PtAAD can specifically select 16:0-ACP substrate to generate 16:1Δ^9^ in chloroplasts when transiently expressed in tobacco leaf tissues.

### Functional Complementation Assay of PtAAD in Yeast Mutant *BY4389*

To further elucidate the catalytic activity of PtAAD enzyme specific for 16:0-ACP, the *PtAAD* gene was expressed in *S. cerevisiae* mutant *BY4389* unable to synthesize unsaturated fatty acids. For this functional assay, positive controls were designed to overexpress either AtFAB2 with 18:0-ACP selectivity or AtAAD3 with 16:0-ACP specificity in *BY4389*. The yeast mutant bearing empty pYES2 vector was used as the negative control. Finally, fatty acid compositions in the transformed *BY4389* cells, wild-type yeast, and negative controls were examined by GC analysis.

**FIGURE 7:**
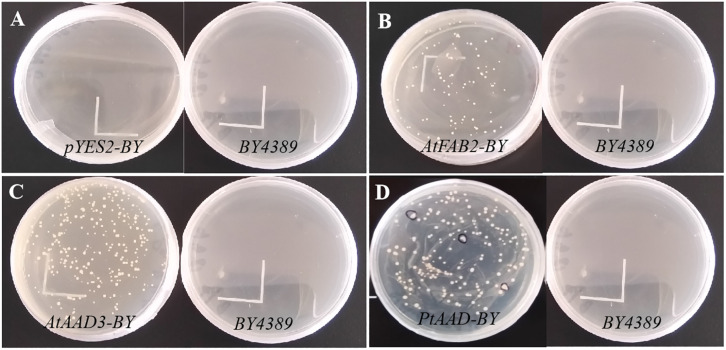
Growth condition of yeast cells in induced medium of 2% galactose (without uracil and any unsaturated fatty acids). **(A)** Mutant yeast of *BY4389* and the transgenic cells expressing pYES2 which were used as blank control. **(B,C)** Mutant yeast of *BY4389* and the transgenic cells expressing *AtFAB2* as well as *AtAAD3* genes which were used as positive controls, respectively. **(D)** Mutant yeast of *BY4389* and the transgenic cells expressing *PtAAD*. The labeling part was used for the subsequent experiment.

**FIGURE 8:**
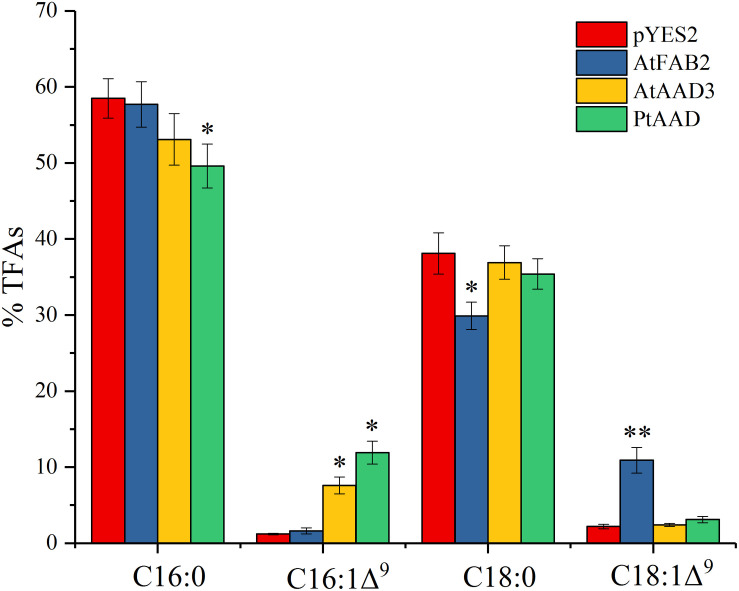
Fatty acid composition in *PtAAD*-expressed yeast mutant *BY4389*. Fatty acids were analyzed by GC and the data are means of mol% ± SE with three biological replicates. “*” and “**” represent statistically significant difference values from empty (pYES2) of *p* < 0.05 and *p* < 0.01 based on two-tailed Student’s *t*-tests, respectively. AtFAB2 and AtAAAD3 were used as positive control and empty vector of pYES2 as negative control. Data are shown as mean ± SE (*n* = 3).

Notably, all transgenic yeasts with exception of pYES2 successfully survived in the selective medium without any uracil and unsaturated fatty acid (UFA), suggesting that the enzyme encoded by *PtAAD* had the activity of acyl-ACP Δ^9^ desaturase that was the same as AtFAB2 and AtAAD3 (Figure 7). FA profiles (Figure 8) revealed that the *PtAAD*-expressed yeast newly produced abundant UFAs despite the uneven level for different UFAs. As expected, *AtAAD3-*transgenic yeast generated more 16:1Δ^9^ (7.6%) than 18:1Δ^9^ (just trace level). Analogously, *PtAAD*-expressed yeast accumulated a high level of 16:1Δ^9^ (11.9%) and a small amount of 18:1Δ^9^. In contrast, *AtFAB2-*expressed yeast produced more 18:1Δ^9^ (10.9%) and less 16:1Δ^9^ (1.6%). The wild-type yeast accumulated 1.2% 16:1Δ^9^ and 2.2% 18:1Δ^9^. Overall, the present data obtained by *in vivo* assays using yeast mutant transformation again revealed that PtAAD had a high substrate specificity for palmitoyl-ACP, thus leading to a significant increase of palmitoleic acid in the yeast cell. Furthermore, this substrate specificity of PtAAD to 16:0-ACP is even higher than the typical 16:0-ACP-specific AtAAD3, indicating that PtAAD can be genetically engineered in other oleaginous organisms for increasing palmitoleic acid production.

Of particular note is that normally, acyl-CoAs but not acyl-A were produced in yeast cells. Our data showed that overexpression of *PtAAD* gene promoted the synthesis and accumulation of palmitoleic acid (16:1Δ^9^) in the yeast, suggesting that PtAAD may use 16:0-CoA as substrate to catalyze synthesis of 16:1Δ^9^. In agreement with our finding, Xue et al. (2016) reported that overexpression of a coccoid green alga acyl-ACP Δ^9^ desaturase gene in the yeast *BY4389* resulted in synthesis and accumulation of oleic acid (18:1Δ^9^), also indicating that this acyl-ACP Δ^9^ desaturase could use acyl-CoA (18:0-CoA) as the substrate to form monounsaturated fatty acid (18:1Δ^9^). Despite no direct evidence showing that acyl-ACP Δ^9^ desaturase can use saturated acyl-CoA as substrate to generate unsaturated fatty acid, these two samples demonstrate that overexpression of an acyl-ACP Δ^9^ desaturase gene in the UFA-deficient yeast mutant *BY4389* indeed led to production of monounsaturated fatty acids (16:1Δ^9^ or 18:1Δ^9^). Collectively, it can be speculated that heterologous acyl-ACP Δ^9^ desaturase could use saturated acyl-CoA as substrate to generate a monounsaturated fatty acid in the yeast cells. The catalytic efficiency of the enzyme may be less using acyl-CoA as substrate instead of acyl-ACP. Functional complementation assay in yeast system can be used to characterize function and substrate specificity of acyl-ACP Δ^9^ desaturase although this is not the best approach.

## Discussion

### *P. tricornutum* Is an Excellent Resource to Produce High-Value ω-7 Palmitoleic Acid

As a kind of seawater microalga, *P. tricornutum* presents multiple advantages: rapid growth, cultivation at commercial scale, strong tolerance for living environment, large biomass productivities, and high oil content (Liang et al., 2014; Caporgno et al., 2016). Therefore, *P. tricornutum* is a promising feedstock for renewable biofuels and food production. Furthermore, its cultivation occupies less farmland compared with traditional oilseed crops, thus solving the problem of insufficient arable land for food and oil production (Srirangan et al., 2008).

Because of high levels of oil accumulation, especially high-value palmitoleic acid, *P. tricornutum* is suitable to commercially and cleanly produce palmitoleic acid with less space and cost to meet huge market demand. Besides, the genome size of *P. tricornutum* is only 27.4 Mb (Bowler et al., 2008), which makes it easier to use this alga as the target for gene functional characterization and metabolic engineering of lipids to produce high-value bioproducts (Cui et al., 2019b). The current findings provide a scientific references for the future improvement of high-quality algae by transgenic or gene editing technology.

### PtAAD With Three Divergent Residues Among the Key Eight Amino Acids in the Conserved Domain Has the Catalytic Activity Specific for 16:0-ACP

Previous reports showed that the eight key amino acids in the function domain of acyl-ACP Δ^9^ desaturases were highly correlated with the substrate specificity of the enzymes (Cahoon et al., 1997; Guy et al., 2011; Troncoso-Ponce et al., 2016). For example, RcSAD1, a typical 18:0-ACP-specific acyl-ACP Δ^9^ desaturase, contains the eight key amino acids (M114, L115, T117, L118, P179, T181, G188, and F189) in the domain (Figure 4 and Table 1). However, Muc-PAD and AtAAD2/3, the three 16:0-ACP-specific AADs, have a few variant amino acids among the eight key amino acids in the domain (Figure 4 and Table 1), with W132 in Muc-PAD, F226/216, S224/214, and T159/149 in AtAAD2/AtAAD3, respectively. Similar to these typical 16:0-ACP-specific AADs, three variant amino acids (F160, A223, and L156, corresponding to L118/156, T181/219, and M114/152 of RcSAD1/AtFAB2, respectively) were also detected in the functional domain of PtAAD, indicating that the three variant amino acids may determine the substrate specificity of PtAAD to 16:0-ACP. Furthermore, these variant AAs in the domain were predicted to locate at the bottom of substrate binding cavity by 3D modeling (Figure 5), offering a suitable space to hold 16:0-ACP rather than 18:0-ACP.

In agreement with these speculations, when the amino acid residues with smaller side chain at certain positions in the domain were changed into those with larger side chain groups of aromatic rings such as tryptophan (Trp, W) and phenylalanine (Phe, F), the catalytic substrate of the enzyme was changed from initial 18:0-ACP to 16:0-ACP (Cahoon et al., 1998). For example, the Muc-PAD only contained one divergent Trp152 (W152) corresponding to L118 in RcSAD1 (Table 1) that exhibited a strong substrate preference for 16:0-ACP but not for 18:0-ACP (Cahoon et al., 1998). Cahoon et al. also reported that when Leu118 in the domain of RcSAD1 was mutated into Trp118, the catalytic activity of the enzyme for 16:0-ACP was significantly increased 80-fold higher than that for 18:0-ACP substrate compared with the wild-type enzyme (Cahoon et al., 1998). In addition, Bryant et al. reported that AtAAD2 and AtAAD3 had F226 and F216 in the domain, respectively (Table 1), and they both showed a strong substrate selectivity for 16:0-ACP (Bryant et al., 2016). The mutated AtFAB2 with single variant F219 in the domain could generate high accumulation of ω-7 fatty acids (16:1Δ^9^ and its extension products) rather than oleic acid (18:1Δ^9^), and the same result was also obtained by two-AA mutants of F219 and S217 in the domain of this acyl-ACP Δ^9^ desaturase (Troncoso-Ponce et al., 2016). Coincidentally, F160 and A223 in the domain of PtAAD may occupy two important positions responsible for the catalytic selectivity of the enzyme because F160 with larger side chain group limits the entrance of 18:0-ACP into the substrate binding cavity, but permits the entrance of 16:0-ACP. To determine whether these three residue changes affect substrate specificity of PtAAD, we created a mutated copy of the *PtAAD* gene (*PtAAD-M*) by direct gene synthesis.

To further examine the enzymatic activity and substrate specificity of PtAAD, we conducted functional analysis by heterologous expression of *PtAAD* gene in *N. benthamiana* leaves and mutant yeast *BY4389*. The *PtAAD*-transient expressed leaves produced a large amount of 16:1Δ^9^ with a significant decrease of 16:0 level compared with the control tobacco leaves (Figure 6). However, when *PtAAD-M* gene was transiently expressed in *N. benthamiana* leaves, it failed to accumulate 16:1Δ^9^, confirming the reliability of 3D modeling prediction. Besides, yeast mutant *BY4389* expressing *PtAAD* restored survival in the selective medium without any unsaturated fatty acid. Also, more importantly, the yeast cells accumulated much higher levels of 16:1Δ^9^, but little 16:0 (Figure 8). Both *in vivo* functional assays evidence that PtAAD has strong enzymatic activity and higher substrate specificity for 16:0-ACP despite no direct evidence obtained to show this substrate specificity of PtAAD. Collectively, our data indicate that PtAAD is the key enzyme responsible for high biosynthesis and accumulation of palmitoleic acid (16:1Δ^9^) in this valued alga. To get the direct evidence showing the substrate specificity of PtAAD, more detailed studies are needed, including prokaryotic expression of this enzyme protein, enzyme protein purification, *in vitro* enzyme activity assays using different acyl-ACP substrates.

### Nitrogen Stress Increases Significant Accumulation of Palmitoleic Acid in *P. tricornutum*

Compared with algal cells in N^+^ group, the relative expression of *PtAAD* gene of alga cells was much higher (around 8 folds) in 1/2N group, leading to the dramatic accumulation of total lipids and 16:1Δ^9^. The excellent fatty acid profile rich in ω-7 palmitoleic acid in the N-stressed algal cells largely increases utilization values of this alga as the desirable feedstock. Moreover, limiting nitrogen supply could be used as an effective way to enhance oil and palmitoleic acid production commercially.

## Conclusion

The present study showed that *P. tricornutum* can produce high levels of monounsaturated palmitoleic acid (16:1Δ^9^), especially in nitrogen-stressed condition. An acyl-ACP Δ^9^ desaturase gene (*PtAAD*) was first identified and cloned from *P. tricornutum*, with upregulated expression under N-stress condition. Moreover, its expression trend was positively related to accumulation pattern of palmitoleic acid in algal cells. Functional assays by transient expression in tobacco leaves and heterologous expression in yeast mutant *BY4389* revealed that PtAAD had a strong enzymatic activity of acyl-ACP Δ^9^ desaturase and higher substrate selectivity for 16:0-ACP instead of 18:0-ACP to produce 16:1Δ^9^, although more studies are needed to get direct evidence. In addition, 3D modeling of PtAAD and protein sequence alignment with other known AADs demonstrated that the key eight amino acids in the functional domain may determine the substrate specificity of AADs. The three variant amino acids F160, A223, and L156 in the domain with F160 located at the bottom of substrate channel may confer the substrate specificity of PtAAD for 16:0-ACP. This structure configuration distinguishes PtAAD from the archetype 18:0-ACP-specific AADs. Taken together, this study brings new insights into the PtAAD-mediated high-level biosynthesis and accumulation of palmitoleic acid in *P. tricornutum*. The identified *PtAAD* gene could be an excellent target for genetic engineering to produce high-value oils enriched with ω-7 FAs in *P. tricornutum* or other oilseed crops for food and industrial applications.

## Data Availability Statement

The original contributions presented in the study are included in the article/Supplementary Material, further inquiries can be directed to the corresponding author/s.

## Author Contributions

BL, JX, and RL designed the experiments and drafted the article. WH did the vector construction, algae strain cultivations, and growth test. BL and YS did fatty acid analysis, material plantation, and data analysis. BL and XW carried out the expression and RNA-seq analysis. RL, XJ, and RM revised the article. All authors contributed to the article and approved the submitted version.

## Conflict of Interest

The authors declare that the research was conducted in the absence of any commercial or financial relationships that could be construed as a potential conflict of interest.

## Footnotes

1 http://genome.jgi.doe.gov/Phatr2/Phatr2.download.html

2 http://smart.embl-heidelberg.de/

3 http://www.ncbi.nlm.nih.gov/Structure/cdd/cdd.shtml

4 http://www.ncbi.nlm.nih.gov/gorf/gorf.html

5 http://gsds.gao-lab.org/

6 http://cn.expasy.org/tools/pi_tool.html

7 http://www.cbs.dtu.dk/services/TargetP/

8 http://www.cbs.dtu.dk/services/ChloroP/

9 http://arabidopsis.org/

10 http://www.ncbi.nlm.nih.gov/

11 https://swissmodel.expasy.org/

## Supplementary Material

Supplementary Figure 1 Fatty acid composition in *N. benthamiana* leaves transiently expressing *PtAAD-M* gene.

Supplementary Figure 2 Subcellular localization of PtAAD:GFP fusion protein in tobacco protoplasts.

Supplementary Table 1 Specific primers used for quantitative RT-PCR and vector construction.

Supplementary Table 2 Fatty acids composition in *P. tricornutum* cultured in N^+^ and 1/2N medium.

Supplementary Table 3 The nucleotide sequence of PtAAD and Phat3_J9316.
