# Supplementary material for: Characterization of a Novel Acyl-ACP Δ9 Desaturase Gene Responsible for Palmitoleic Acid Accumulation in a Diatom Phaeodactylum tricornutum
Source: Front Microbiol. 2020 Dec 16;11:584589. doi: 10.3389/fmicb.2020.584589 (PMC7772203; doi:10.3389/fmicb.2020.584589)
Supplement: Supplementary Figure 1 — Fatty acid composition in N. benthamiana leaves transiently expressing PtAAD-M gene. [file Data_Sheet_1.pdf]

Supplementary Material

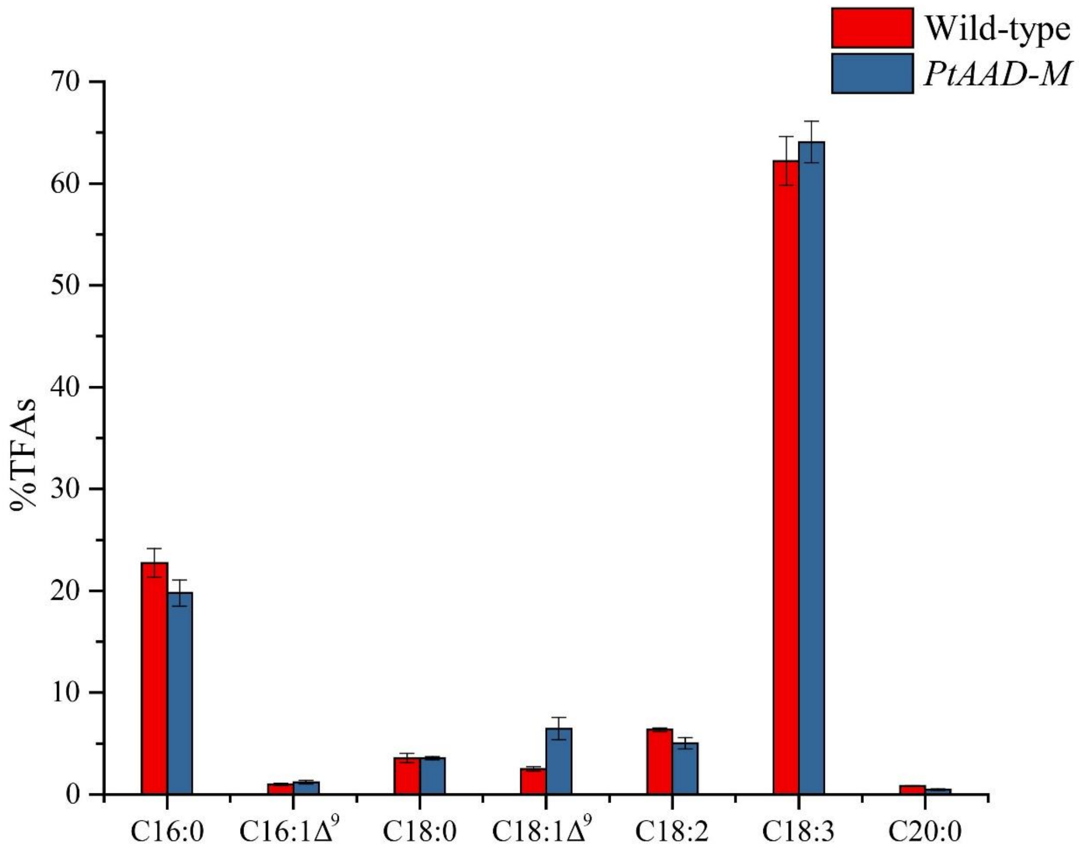

Supplementary FIGURE S1 | Fatty acid composition in *N. benthamiana* leaves transiently expressing *PtAAD-M* gene. Fatty acids were analyzed by GC and the data are means of Mol% ± SE with three independent replicates. Data are showed as mean ± SE (n = 3).

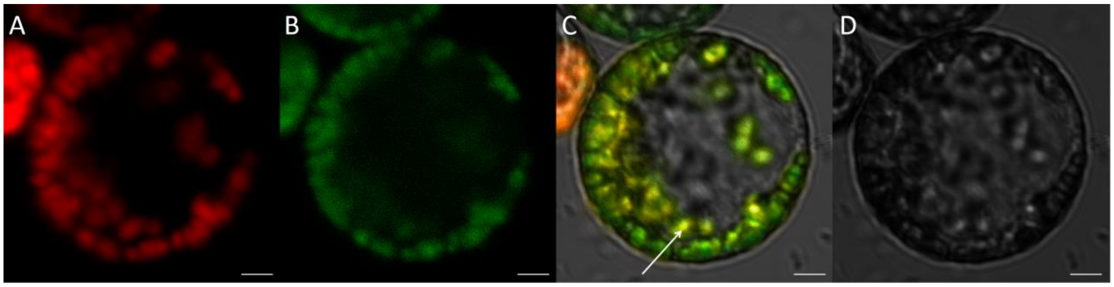

Supplementary FIGURE S2 | Subcellular localization of PtAAD:GFP fusion protein in tobacco protoplasts. A laser scanning confocal microscope (DeltaVision Olympus) was used to obtain these images. (A) Red spontaneous fluorescence of chloroplast; (B) Green fluorescence of PtAAD:GFP fusion protein; (C) Yellow overlapping fluorescence of PtAAD:GFP fusion protein and chloroplast; (D) Protoplast cells under visible light. Bar=50 μm.

Supplementary TABLE 1 | Primers used in this study

Name	Sequence (5'→3')	Purpose
Ptactin-F	AGGCAAAGCGTGGTGTTCTTA	Internal reference
Ptactin-R	TCTGGGGAGCCTCAGTCAATA	
PtAAD-1F	TGATGCGTGGACAGATTG	qPCR analysis
PtAAD-1R	AGGTACTCCCGTTCCTTTT	
PtAAD-2F	TTCTCCGCTTCACAACTC	Detection analysis
PtAAD-2R	ACAATCTGTCCACGCATC	
p1303-PtAAD-F	***Pst*I** **CG**GAATTCATGTTGTCTACGAAACTTTTCTGG	Cloning for pCAM1303
p1303-PtAAD-R	***Kpn*I** **CG**GAATTCTCAAGCCATCCGACCATAAATCCA	
pYES2-PtAAD-F	***EcoR*I** **CG**GAATTCATGTTGTCTACGAAACTTTTCTGG	Cloning for pYES2
pYES2-PtAAD-R	***Xba*I** **GC**TCTAGATCAAGCCATCCGACCATAAATCCA	

Supplementary TABLE 2 | Fatty acids composition in P. tricornaturn cultured in N+ and 1/2N medium

Main fatty acids（% TFAs）	Cultured Days				
	0	5		10	
	N^+	N^+	1/2 N	N^+	1/2 N
14:00	6.33±0.33	6.01±0.21	4.96±0.25	6.18±0.34	4.34±0.17
16:00	17.82±1.43	14.27±1.12	27.75±1.70	15.12±1.13	31.43±1.44
18:00	4.71±0.12	1.17±0.07	1.38±0.08	1.85±0.07	1.42±0.06
20:00	0.35±0.03	0.16±0.02	0.12±0.01	0.13±0.01	0.11±0.01
22:00	0.31±0.02	0.21±0.02	0.19±0.01	0.18±0.01	0.2±0.02
$16:1\Delta^{9}$	25.83±1.05	32.74±1.27	40.25±2.15	33.56±1.37	44.61±2.42
$18:1\Delta^{9}$	2.98±0.10	2.77±0.12	3.18±0.11	2.93±0.08	3.57±0.14
$18:1\Delta^{11}$	1.76±0.02	0.89±0.04	2.02±0.08	0.62±0.03	2.99±0.11
20:01	0.65±0.03	0.25±0.02	0.12±0.01	0.11±0.01	0.13±0.01
16:2 n-4	2.18±0.14	2.35±0.09	0.56±0.03	2.67±0.11	0.43±0.02
16:3 n-4	4.7±0.12	6.76±0.1	1.31±0.07	5.18±0.16	1.12±0.06
18:2 n-6	2.66±0.1	1.84±0.06	1.42±0.07	2.28±0.11	0.72±0.05
18:3n-6	1.24±0.1	0.51±0.03	0.92±0.08	0.39±0.03	0.75±0.04
18:3n-3	3.1±0.11	1.08±0.08	0.54±0.02	1.57±0.09	0.33±0.03
C20:5n-3(EPA)	20.78±0.75	24.3±0.97	11.05±0.31	23.27±1.3	5.28±0.42
C22:6n-3(DHA)	2.1±0.06	1.79±0.12	1.73±0.05	1.85±0.1	0.51±0.03
Others	2.62±0.47	2.9±0.45	2.4±0.32	2.11±0.37	2.06±0.42
SFAs	28.52	21.82	34.4	23.46	37.5
MUFAs	30.32	36.65	45.67	37.22	51.3
PUFAs	36.54	38.63	17.53	37.21	9.14
ω–7 FAs	26.69	33.63	42.27	34.18	47.6

Note: %TFAs means % of total fatty acids. Data are counted and calculated as means ± SE of three biological repetitions.

Supplementary TABLE S3 | The nucleotide sequence of PtAAD and Phat3_J9316

Phat3_J9316	>Phat3_J9316<br><br>GAAAATGCTTGGCAGCCGCAAGATTACCTTCCCGATCTCT<br>CGCAGGACAATTGGCATGACTCCATCAAGGAAGTCCGTG<br>CCATGGCCAAGGAAATTCCGGACGAACTCTTGGTCGTGT<br>TGATTGGCGATATGGTGACGGAAGAAGCTCTCCCAACCT<br>ACCAGACGCTCTTGAATACCTTTGAAGGTTGCGATGACCC<br>CACCGGCACTAGTGAATCTCCCTGGGCACGCTGGTCCCG<br>CGGTTGGACTTCGGAAGAAAACCGGCACGGCGATTTGCT<br>CAACAAATATCTCTATCTGGGCGGCCGTTGCGACATGCG<br>CAACATCGAGGTCACCATCCAGCACTTGATTACGAACGG<br>ATTCAACCCGCAAGCCCGCAAAGACCCCTACCGCGGATT<br>CGTGTATACGTCCTTTCAGGAACGTGCGACCAAAATCTCG<br>CACGGCAACGTGGGCAAGCTGGCCCGCACGTACGGCGAA<br>AAGAATCTGAACAAGATTTGTGCAAAGATCGCGGGCGAC<br>GAAGGTCGCCACGAGAAGGCTTATCAAATCTTTTCCGAA<br>GAAATCTTAAAGCGTGATCCGGACGGACTCATTCACGTTT<br>TTGGTGACATGATGCGTGGACAGATTGTCATGCCTGCGG<br>AACAGATGACGGATGGCAAAGATCCGGATTTGTACGATA<br>ACTTTTCCATGGTTGCCCAGAAGACGGGTGTGTATACAGC<br>CCTCGATTACGCAGAAATCATCGACCATCTGGTGAAACG<br>GTGGGATTTGGAGCACCTGGAAGGTCTGTCACCGGCTGC<br>CGAAAAGGAACGGGAGTACCTGTGCCGATTGCCGGAACG<br>CTATCGCAAATTGGCGACCCGATCGATGAACAAAAAGAA<br>GAAAGTTACCGAAGACGAGGATCCACTCAAATCCTTTGG<br>CTGGATTTATGGTCGGATGGCTTGA
PtAAD	>PtAAD<br><br>ATGTTGTCTACGAAACTTTTCTGGACGAGTTCGGTTTTGG<br>CTTCCAGTGCGGTGCTTGCCTTTCCCACGTCGCCTGCGAC<br>TAGGACTCCTCGCAGCACTTCCATTCTGAAGGTTGCCGTG<br>GACCCCACAACAGTGACCAAGAAGGAATACGAAGACAT<br>CTGCGGGGTTTCGTTCGACGCCGACACGTTGGAAGATCG<br>TCTAAAGGCAACGAACTACTTGTATCCCAAACACGTCGA<br>AGTCATCGATGACATTGCTCCTATTGCCGGAGCCATGGTG<br>GATGAAATTCTCTTGGAAACTGGTGAAAATGCTTGGCAG<br>CCGCAAGATTACCTTCCCGATCTCTCGCAGGACAATTGGC<br>ATGACTCCATCAAGGAAGTCCGTGCCATGGCCAAGGAAA<br>TTCCGGACGAACTCTTGGTCGTGTTGATTGGCGATATGGT<br>GACGGAAGAAGCTCTCCCAACCTACCAGACGCTCTTGAA<br>TACCTTTGAAGGTTGCGATGACCCCACCGGCACTAGTGA<br>ATCTCCCTGGGCACGCTGGTCCCGCGGTTGGACTTCGGAA<br>GAAAACCGGCACGGCGATTTGCTCAACAAATATCTCTAT

	CTGGGCGGCCGTTGCGACATGCGCAACATCGAGGTCACC<br>ATCCAGCACTTGATTACGAACGGATTCAACCCGCAAGCC<br>CGCAAAGACCCCTACCGCGGATTCGTGTATACGTCCTTTC<br>AGGAACGTGCGACCAAAATCTCGCACGGCAACGTGGGCA<br>AGCTGGCCCGCACGTACGGCGAAAAGAATCTGAACAAGA<br>TTTGTGCAAAGATCGCGGGCGACGAAGGTCGCCACGAGA<br>AGGCTTATCAAATCTTTTCCGAAGAAATCTTAAAGCGTGA<br>TCCGGACGGACTCATTCACGTTTTTGGTGACATGATGCGT<br>GGACAGATTGTCATGCCTGCGGAACAGATGACGGATGGC<br>AAAGATCCGGATTTGTACGATAACTTTTCCATGGTTGCCC<br>AGAAGACGGGTGTGTATACAGCCCTCGATTACGCAGAAA<br>TCATCGACCATCTGGTGAAACGGTGGGATTTGGAGCACC<br>TGGAAGGTCTGTCACCGGCTGCCGAAAAGGAACGGGAGT<br>ACCTGTGCCGATTGCCGGAACGCTATCGCAAATTGGCGA<br>CCCGATCGATGAACAAAAAGAAGAAAGTTACCGAAGAC<br>GAGGATCCACTCAAATCCTTTGGCTGGATTTATGGTCGGA<br>TGGCTTGA
